# Enhanced antitumoral efficacy and immune response following conditionally replicative adenovirus containing constitutive HSF1 delivery to rodent tumors

**DOI:** 10.1186/1479-5876-10-101

**Published:** 2012-05-21

**Authors:** Rong Fan, Cheng Wang, Yang Wang, Ping Ren, Pingping Gan, Hui Ji, Zian Xia, Suiyu Hu, Qiongyao Zeng, Wei Huang, Yebin Jiang, Xi Huang

**Affiliations:** 1 Department of Integrative Medicine of Traditional Chinese Medicine and Western Medicine, Xiangya Hospital, Central South University, Changsha, Hunan, 410008, China; 2 Institute of Translational Medicine and Therapeutics, School of Medicine, University of Pennsylvania, Philadelphia, PA, 19104, USA; 3 Department of Oncology, Xiangya Hospital, Central South University, Changsha, Hunan, 410008, China; 4 Osteoporosis and Arthritis Lab, Department of Radiology, University of Michigan, Ann Arbor, MI, 48109, USA

**Keywords:** HSF1, Cancer immunotherapy, Oncolytic adenovirus

## Abstract

**Background:**

Oncolytic adenoviruses are promising as anticancer agents but have limited clinical responses. Our previous study showed that heat shock transcription factor 1 (HSF1) overexpression could increase the anti-tumor efficacy of E1B55kD deleted oncolytic adenovirus through increasing the viral burst. Due to the important roles of heat shock proteins (HSPs) in eliciting innate and adaptive immunity, we reasoned that besides increasing the viral burst, HSF1 may also play a role in increasing tumor specific immune response.

**Methods:**

In the present study, intra-dermal murine models of melanoma (B16) and colorectal carcinoma (CT26) were treated with E1B55kD deleted oncolytic adenovirus Adel55 or Adel55 incorporated with cHSF1, HSF1i, HSP70, or HSP90 by intra-tumoral injection. Tumors were surgically excised 72 h post injection and animals were analyzed for tumor resistance and survival rate.

**Results:**

Approximately 95% of animals in the Adel55-cHSF1 treated group showed sustained resistance upon re-challenge with autologous tumor cells, but not in PBS, Adel55, or Adel55-HSF1i treated groups. Only 50–65% animals in the Adel55-HSP70 and Adel55-HSP90 treated group showed tumor resistance. Tumor resistance was associated with development of tumor type specific cellular immune responses. Adel55-cHSF1 treatment also showed higher efficacy in diminishing progression of the secondary tumor focus than Adel55-HSP70 or Adel55-HSP90 treatment.

**Conclusions:**

Besides by increasing its burst in tumor cells, cHSF1 could also augment the potential of E1B55kD deleted oncolytic adenovirus by increasing the tumor-specific immune response, which is beneficial to prevent tumor recurrence. cHSF1 is a better gene for neoadjuvant immunotherapy than other heat shock protein genes.

## Background

Malignant tumors are of a highly complex nature, a cure will probably result only from complete eradication of all of the tumor cells. Current modalities for cancer therapy are not selective and may have severe adverse effects. Oncolytic adenoviruses are a class of promising anticancer agents, which are designed to selectively replicate in tumor cells and lead to cancer-specific cytotoxicity [1,2]. However, clinical experiences show that these agents alone failed to generate sustained clinical responses or to cause complete tumor regressions [3]. Our previous study showed that constitutively active heat shock transcription factor 1 (cHSF1) expression could dramatically increase the replication of the replication competent adenovirus Adel55 in tumor cells and enhance the antitumor efficacy of Adel55 in vitro and in vivo [4]. Increasing evidence indicates that heat shock proteins (HSPs), which are transcribed by HSF1 play important roles in eliciting innate and adaptive immunity [5-7]. We reasoned that besides increasing the viral burst, cHSF1 may also play a role in increasing tumor specific immune response.

The ability of HSPs to chaperone peptides and to promote T-cell responses by activating dendritic cells (DCs) seems to be the common feature associated with all the major HSPs, including HSP110, gp96, HSP90, and HSP70 [8-11]. When peptide-loaded HSPs are released from the cells, they bind to antigen-presenting cells (APCs) mainly through the CD91 receptor (a2-macroglobulin receptor), CD40 and LOX-1. Once taken up by APCs, the peptides are translocated to MHC class-I molecules, which traffic to the cell surface enabling efficient presentation to CD8+ T cells, a vital step in raising a systemic antitumor response. Generating antitumor immune responses against tumor-associated self-antigens and mutated antigens requires endogenous nonmicrobial danger molecules that activate APCs. Besides their ability to chaperone antigenic peptides, HSPs may function as endogenous nonmicrobial danger molecules [12-14] that can up-regulate the expression of costimulatory and antigen-presenting molecules on DCs. They also stimulate the secretion of proinflammatory cytokines such as interleukin 12 (IL-12), leading to the activation of natural killer (NK) cells and other immune cells [15]. Tumor-derived HSPs provide effective tumor vaccines in mouse models [16-18], and the ability of human melanoma-derived HSP70 to stimulate autologous melanoma-specific T cells also has been shown *in vitro*[19].

Based on these evidences, we hypothesized that cHSF1 overexpression could achieve more potent tumor immunogenicity than any HSP alone due to its ability to increase various HSPs expression at the same time. We evaluated the effect of enhanced tumor immunogenicity by direct intra-tumoral injection of Adel55-cHSF1 or control viruses prior to surgical excision of intra-dermal murine tumors to result in measurable anti-tumor immune responses. We further evaluated the ability of Adel55-cHSF1 to alter growth of alternative tumor foci and on survival of animals.

## Materials and methods

### Plasmids

The E1B55kD gene deleted oncolytic adenovirus vector pAdel55 was established by nested PCR using pXC1 (Microbix Biosystems, Ontario, Canada) as the template. The viral region comprising nucleotides 1318–2038 was amplified using a primer set of 5 GCC GAC ATC ACC TGT GTC TAG AGA ATG -3′ (L1) and 5′- TCA GAT GGG TTT CTT CAC TCC ATT TAT CCT-3′ (R1). The region containing nucleotides 2005–2266 was amplified with another primer set of 5′-ATA AAG GAT AAA TGG AGT GAA GAA ACC CAT CTG AG-3′ (L2) and 5′-GAA GAT CTA TAC AGT TAA GCC ACC TAT ACA ACA-3′ (R2). Using the mixture of the two PCR products as template, a 955 bp fragment was then amplified using primers L1 and R2. This fragment was cut by Xba I and Bgl II and cloned into pXC1 to generate the plasmid pXC1-del55. SV40 polyA (160 bp) were obtained by PCR using pcDNA3 (invitrogen) as template and two primers: 5′-TGT GGA TCC TCT AGA GCT CGC TGA-3′ and 5′-TCT AGA TCT CGA GCC CCA GCT GGT-3′. Then it was digested with BamH I and Bgl II and cloned into the Bgl II site of pXC1- del55 to generate the plasmid pAdel55. The correct construction of this vector was confirmed by DNA sequencing. Constitutively active heat shock transcription factor 1 (cHSF1) and HSF1 miRNA inhibitor (HSF1i) gene were made as described previously [4]. First, cHSF1, HSF1i, HSP70 or HSP90 gene was cloned into the polycloning site of shuttle vector pCA13 (Microbix Biosystems, Ontario, Canada). Then the whole gene expression cassette containing CMV promoter, transgene and SV40 polyA site was cut from pCA13-cHSF1, pCA13-HSF1i, pCA13-HSP70 or pCA13-HSP90 by Bgl II and subcloned into the corresponding site of pAdel55 to generate pAdel55-cHSF1, pAdel55-HSF1i, pAdel55-HSP70 or pAdel55-HSP90.

### Virus construction

Adenovirus was generated by standard homologous recombination techniques using the plasmid pAdel55, pAdel55-cHSF1, pAdel55-HSF1i, pAdel55-HSP70 or pAdel55-HSP90 and the adenovirus packaging plasmid pBHGE3 (adenovirus packaging plasmid, Microbix Biosystems, Ontario, Canada) in HEK293 cells. Recombinant adenovirus plaque purified, propagated on HEK293 cells and purified by CsCl gradient according to standard techniques. Particle titers of all adenoviruses were determined by absorbance measurements at 260 nm, and functional plaque formation unit (pfu) titers were determined by plaque assay on HEK293 cells. The titer of stocks was 2.3 × 10^11^ pfu/ml for Adel55 (bioactivity 25.1), 5.1 × 10^11^ pfu/ml (bioactivity 23.5) for Adel55-cHSF1, 3.9 × 10^11^ pfu/ml (bioactivity 22.8) for Adel55-HSF1i, 2.9 × 10^11^ pfu/ml (bioactivity 23.3) for Adel55-HSP70, and 4.1 × 10^11^ pfu/ml (bioactivity 24.3) for Adel55-HSP90.

### Cell lines and culture

C57B/6 mouse origin (H-2b) melanoma cell line B16 and Balb/C origin (H-2D) colorectal tumor cell line CT26 were obtained from American Type Tissue Collection (ATCC, Rockville, MD, USA). Cells were kept at 37 degree, 5% CO_2_ and 95% humidity in Dulbecco’s modified eagle medium (Cellgro, Herndon, VA, USA) supplemented with 10% (v/v) heat inactivated fetal bovine serum (Invitrogen, Carlsbad, CA, USA), 2 mM L-glutamine and 100 units/ml penicillin and 1,000 μg/ml streptomycin (Invitrogen, Carlsbad, CA, USA).

### Animal experiments

All animal experiments were carried out in accordance with the National Institute of Health Guide for the Care and Use of Laboratory Animals. Five-week-old female C57B/6 or Balb/C mice were injected intradermally with 1 × 10^6^ B16 or CT26 cells in a total volume of 50 μl by aseptic technique on the murine dorsum after shaving in sterile phosphate buffered saline. Tumor growth was measured with calipers. The perpendicular tumor diameter was measured every 5 days and tumor volume (V) was calculated by the formula for a rotational ellipsoid: V (mm^3^) = length × width^2^/2. In our multifocal tumor model, an initial inoculum was given on the left dorsum. On day 5, a secondary inoculum of tumor cells was transplanted on the opposite flank.

Animals were injected with 5 × 10^8^ pfu Adel55, Adel55-cHSF1, Adel55-HSF1i, Adel55-HSP70, Adel55-HSP90 or phosphate buffered saline once a day for 2 consecutive days. Tumors were surgically excised 72 h later with small margins and the skin sutured using 3.0 nylon.

### CTL assay

Cytotoxicity was assessed by the ability of spleen effector cells to lyse various tumor target cells. Splenocytes were derived by mechanical disruption of spleens under aseptic conditions in PBS. Red blood cells were removed by 5-min incubation in ammonium chloride lysis buffer (Pharmingen). Splenocytes were stimulated for 6 days in 24-well tissue culture plates at a ratio of 150:1 with 25 μg/ml mitomycin C-treated homolog cells in RPMI medium containing 10% fetal calf serum (RPMI-10% FCS) supplemented with 1 ng/ml recombinant murine IL-2 (BD Biosciences). Splenocytes were harvested from plates by Ficoll density centrifugation and added to 96- well U-bottom plates (Corning) in RPMI- 10% FCS. Target cells were harvested, washed in PBS and labeled with 200 uCi of 51Cr (Na_2_CrO_4_ in sterile saline; Amersham Biochemicals) in DMEM-10% FCS supplemented with 50 μM 2-mercaptoethanol for 90 min. Target cells were washed three times with ten times the volume of PBS and then added to plates containing splenocytes at the ratios described in the figure legends. After 4 h incubation at 37 degree, 5% CO_2_, the plates were harvested using a Skatron Harvesting System (Skatron). Chromium release into supernatant was counted using an ICN gamma counter. Spontaneous Cr leakage was measured by six wells that did not contain splenic effector cells. Maximum Cr leakage was determined by addition of Triton X-100 to a final concentration of 0.8% to six wells. Corrected % lysis was determined by the following formula: Corrected lysis = 100 × (effector cell sample chromium release - spontaneous target chromium released)/(maximum target chromium released - spontaneous target chromium released).

### Statistical analysis

Data are expressed as means ± SD values. Student’s *t* test was applied to study the relationship between the different variables. Statistical significance was taken at *P* < 0.05. Tumor resistance statistics was determined by Fisher exact analysis. Survival analysis was performed using the Kaplan-Meier test.

## Results

### Intratumor injection of Adel55-cHSF1 prevents the growth of Secondary Tumor

On the basis of our previous study, we applied Adel55-cHSF1 for tumor gene therapy in immune-competent mice. It has been reported that HSPs overexpression could increase the tumor specific immune response [8-11]. Since we overexpressed cHSF1 by using Adel55-cHSF1, we reasoned that Adel55-cHSF1 might also be able to increase the immune response. C57B/6 or Balb/C mice were injected intradermally with 1 × 10^6^ B16 or CT26 cells, and then mice with approximately 100 mm^3^ B16 or CT26 tumors growing subcutaneously were treated by intratumoral administration of 5 × 10^8^ pfu different recombinant adenoviruses or vehicle control once a day for 2 consecutive days, and then surgically excised the tumor after 72 h. Animals were given a 2-week recovery period and then injected on the opposite dorsal surface with autologous tumor cells. Positive tumor formation was determined by at least minimal, palpable tumor 2 weeks from the time of tumor injection.

We show in Table 1 the number of animals resistant to tumor formation in the various treatment groups. Of note, no animals had recurrent tumor formation at the initial surgical site. Although tumor resistance was observed in the Adel55-cHSF1, Adel55-HSP70 and Adel55-HSP90 treatment group, Adel55-cHSF1 was the most effective one with 95% animals resistant to tumor re-innoculation, while Adel55-HSP70 or Adel55-HSP90 only resulted in 50–65% animals free of tumors (*P* < 0.05, Adel55-cHSF1 vs. Adel55-HSP70 or Adel55-HSP90). No tumor resistance was detected in the PBS, Adel55 or Adel55-HSF1i injected groups. Similar results were got from both B16 and CT26 groups.

**Table 1:** Number of animals resistant to tumor formation after treatment

	***PBS***	***Adel55***	***Adel55-cHSF1***	***Adel55-HSF1i***	***Adel55-HSP70***	***Adel55-HSP90***
B16	0/21	0/19	20/21*	0/21	12/21	11/21
CT26	0/22	0/17	24/25*	0/23	15/23	14/23

Mice with approximately 100 mm^3^ B16 or CT26 tumors growing subcutaneously were treated by intratumoral administration of 5 × 10^8^ pfu agents in the top column once a day for 2 consecutive days. Tumors were excised after 72 h and animals were re-inoculated with autologous tumor cells 2 weeks after surgery. Positive tumor formation was determined by at least minimal, palpable tumor 2 weeks from the time of tumor injection. Untreated animals as a positive control were injected and showed tumor formation. *represent statistical significance (*P* < 0.05, Adel55-cHSF1 vs. Adel55-HSP70 or Adel55-HSP90).

### Adel55-cHSF1 can induce tumor type specific cytotoxic T lymphocyte activity when splenocytes are stimulated in vitro

**Figure 1:**
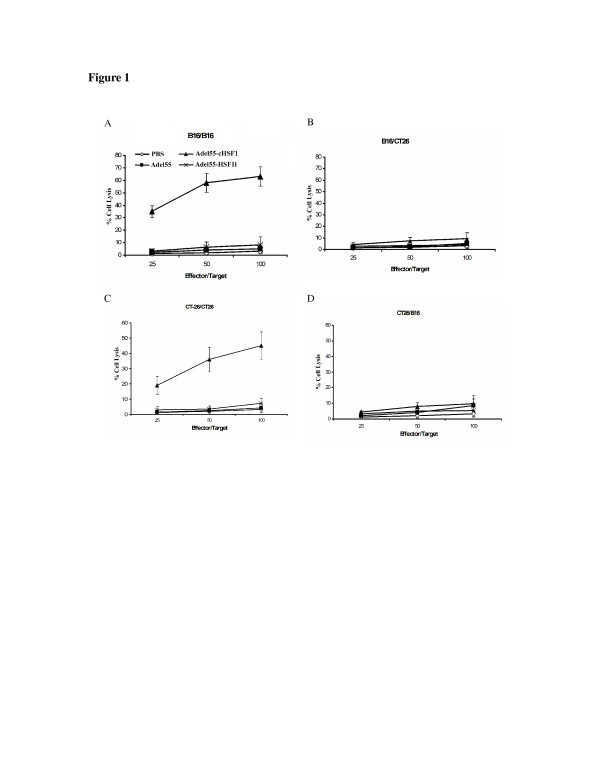
**Tumor type specific cytotoxic T lymphocyte activity induced by Adel55-cHSF1 administration.** Established subcutaneously B16 tumors were treated by intratumoral injection of PBS, or 5 × 10^8^ pfu Adel55, Adel55-cHSF1 or Adel55-HSF1i. Splenocytes from resistant and non-resistant animals were obtained two weeks after the second tumor challenge and restimulated with mitomycin C-treated B16 cells as effector cells. The effector cells were then assayed for cytolytic function by using B16 (**A**) or CT26 (**B**) cells as targets. The ratio 25, 50 and 100 of effector/target cells were detected. Percent specific lysis of target cells is depicted on the y-axis. All results are shown as mean ± SD (n = 5). Similarly, established subcutaneously CT26 tumors were treated by intratumoral injection of PBS, or 5 × 10^8^ pfu Adel55, Adel55-cHSF1 or Adel55-HSF1i. Splenocytes from resistant and non-resistant animals were obtained two weeks after the second tumor challenge and restimulated with mitomycin C-treated CT26 cells as effector cells. The effector cells were then assayed for cytolytic function by using CT26 (**C**) or B16 (**D**) cells as targets.

Next, we determined the immunological basis of tumor resistance in these animals. In previous experiments, cellular immune responses have been detected which can confer tumor immunity to the various cell lines utilized [20-22]. Established subcutaneously tumors were treated by intratumoral injection of PBS, Adel55, Adel55-cHSF1 or Adel55-HSF1i. Splenocytes from resistant and non-resistant animals were obtained two weeks after the second tumor challenge. Spleen cells were restimulated with mitomycin C-treated homolog cells to get effector cells. The effector cells were then assayed for cytolytic function by using B16 or CT26 cells as targets. As shown in Figure 1, B16 tumor resistant animals had developed B16 specific cellular immune responses (Figure 1A and 1B), and CT26 tumor resistant animals had developed CT26 specific cellular immune responses (Figure 1C and 1D). In contrast, animals injected with PBS, Adel55, or Adel55-HSF1i showed lack of cell specific cellular immunity. This indicates that Adel55-cHSF1 can induce tumor type specific cytotoxic T lymphocyte activity when splenocytes are stimulated in vitro. Results were similar from splenocytes of Adel55-HSP70 or Adel55-HSP90 treated animals which is consistant with the previous studies (data not shown). This experiment indicates that cHSF1 delivery can lead to tumor resistance due to induction of tumor type specific cytotoxic T lymphocyte.

**Figure 2:**
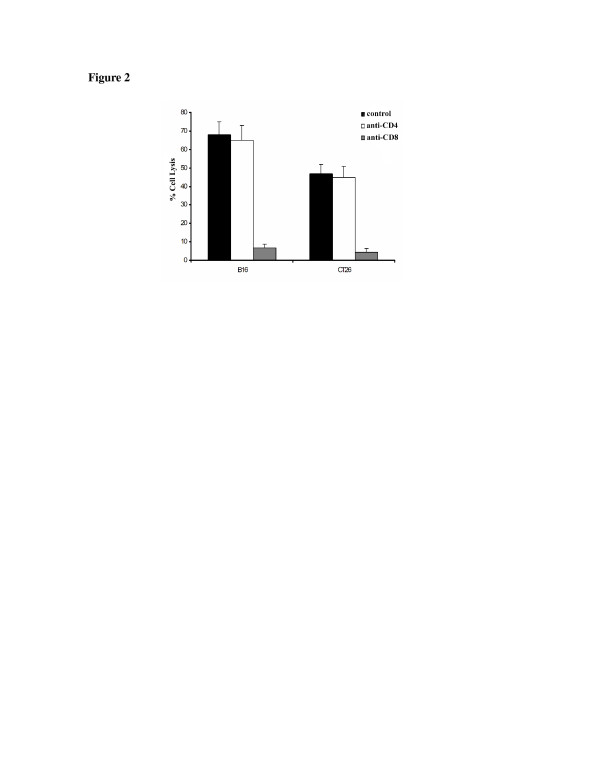
**Cytotoxic T lymphocyte assays with blocking antibodies.** Percent specific lysis of target cells is depicted on the y-axis. Pooled splenocytes from two animals resistant to autologous tumor challenge from the treatment groups identified on the x-axis at an effector to target ratio 100:1. Anti-CD4 or anti-CD8 antibodies (Pharmingen) were added at 30 μL/well to inhibit activity. The data represent the mean ± SD of three determinations.

To detect what type of cytotoxic T cells induced by Adel55-cHSF1, cytotoxic T lymphocyte assays with blocking antibodies was performed. As shown in Figure 2, only when anti-CD8 antibody was added, the tumor type specific cytotoxic T lymphocyte activity could be inhibited. It indicates that these types of T cells are CD8 type.

### Adel55-cHSF1 can increase the overall survival of animals re-inoculated with secondary tumors

**Figure 3:**
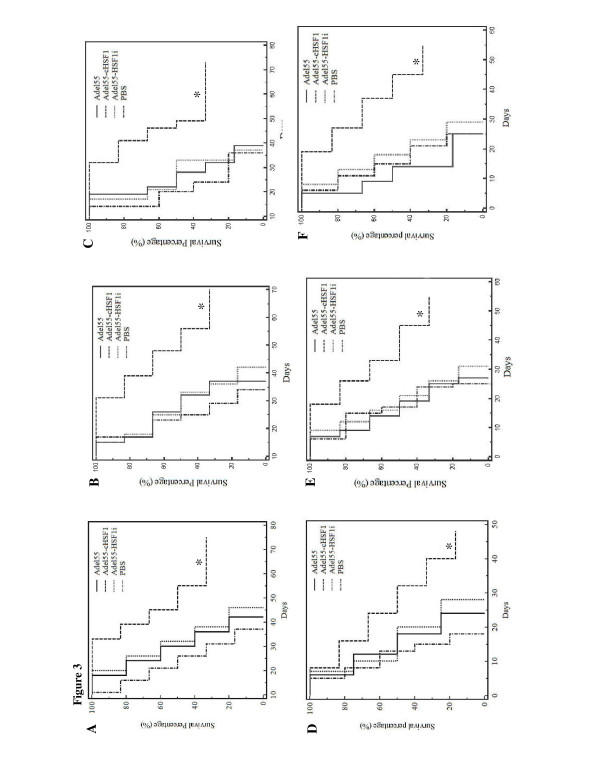
**Kaplan–Meier survival analysis of mice after re-challenging with autologous tumor.** C57B/6 or Balb/C mice with approximately 100 mm^3^ B16 (**A**, **B**, **C**) or CT26 tumors (**D**, **E**, **F**) growing subcutaneously were treated by intratumoral administration of 5 × 10^8^ pfu of PBS, Adel55, Adel55-cHSF1 or Adel55-HSF1i once a day for 2 consecutive days. Tumors were excised after 72 h. Animals were re-inoculated with autologous tumor cells subcutaneously 2 weeks after surgery and followed for survival. Criteria for removal of animals from consideration included weight loss > 12%, inability to ambulate, inability to feed, or loss of normal grooming. Data shown are from three independent experiments for both tumor models (n = 4-6 for all groups). (*) denotes statistical significance (Adel55-cHSF1 vs. PBS or Adel55 or Adel55-HSF1i).

Next we addressed the effect of Adel55-cHSF1 neoadjuvant therapy on overall survival of animals. In our experience, surgical excision of tumors less than 200 mm^3^ did not result in metastasis or affect survival. Local or distant tumor relapse was not observed after surgical excision of primary tumors in any of the models studied. Overall survival of animals after neoadjuvant adenovirus therapy is shown in Figure 3, for the B16 (Figure 3v, B, C) and CT26 (Figure 3D, E, F) tumor models, respectively. Data shown are from three independent experiments. We observed a long-term survival rate in the Adel55-cHSF1-treated group, whereas no mouse survived in the PBS-, Adel55- or Adel55-HSF1i-treated groups at the end of the experiments (*P* < 0.05). In Figure 3D, one mouse treated with Adel55-cHSF1 died at 1 week due to unknown illness, with no palpable tumor formation, while in Figure 3E and F, no mice treated with Adel55-cHSF1 died until around two to three weeks, which made the long term survival rate in Figure 3D less than in the other two experiments. We didn’t find correlation between the illness and the Adel55-cHSF1 injection.

In both tumor models, survival advantage is associated with Adel55-cHSF1 treatment. PBS, Adel55 or Adel55-HSF1i intratumor injections prior to surgical excision did not confer any protection against secondary tumor challenge or prolongation of survival. All animals which formed persistent tumors upon re-inoculation of tumor cells eventually died. In the Adel55-cHSF1 group, some animals did form palpable tumors by day 7 which subsequently regressed. These animals remained tumor free for the duration of the study. The survival benefit associated with Adel55-cHSF1 treatment might be related to development of anti-cellular immunity as described above. Both B16 and CT26 are aggressive tumors and no late regressions were observed.

### Adel55-cHSF1 can prevent micrometastases of tumors and is superior to Adel55-HSP70 or Adel55-HSP90

**Figure 4:**
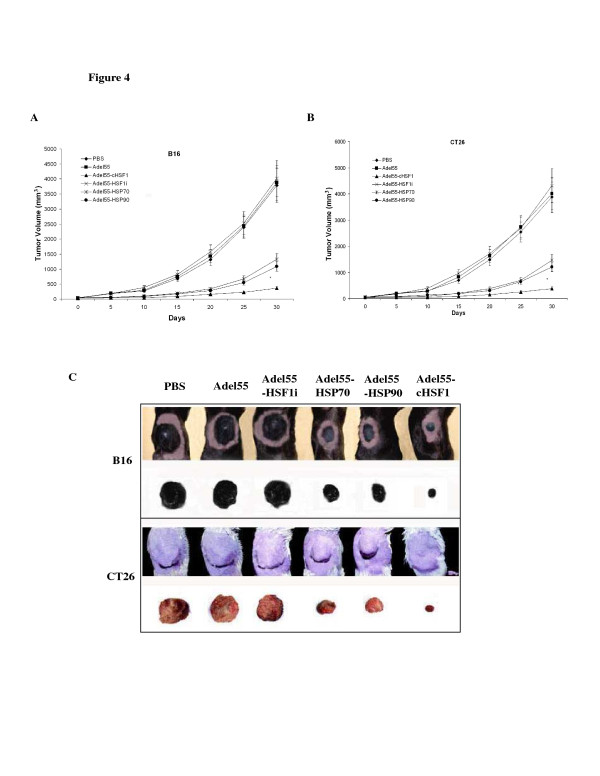
**Effect of neoadjuvant immuno-gene therapy on distant tumor growth.** (**A**) Mice were inoculated with tumor cells on one flank and 5 days later on the opposite flank. The perpendicular tumor diameter was measured every 5 days, and tumor volume (V) was calculated by the formula for a rotational ellipsoid: V (mm^3^) = length × width^2^/2. When the opposite tumor foci reached 50 mm^3^, the larger tumor was injected with PBS, 5 × 10^8^ pfu Adel55, Adel55-cHSF1, Adel55-HSF1i, Adel55-HSP70 or Adel55-HSP90 and surgically excised 72 h post injection. The progression of the opposite tumor foci was measured for one month. (**A**) C57B/6 mice inoculated with B16 tumor cells (n = 8). (**B**) Balb/C mice inoculated with CT26 tumor cells (n = 8). *represent statistical significance (*P* < 0.05, Adel55-cHSF1 vs. Adel55-HSP70 or Adel55-HSP90). (**C**) The picture shows the opposite tumor foci of different groups of Balb/C or C57B/6 mice bearing B16 or CT26 tumors and tumors dissected at day 30.

Tumor relapse is one of the difficulties with surgical therapy for many different tumors, which is believed to be a consequence of micrometastatic tumor not previously detected. Previous studies demonstrated that HSP gene delivery could prevent the tumor micrometastases. To compare the effect of cHSF1 with HSP gene, we treat C57B/6 or Balb/C mice with multifocal B16 or CT26 tumor which is clinically applicable model of micrometastases. Only a single tumor focus was treated with PBS, Adel55, Adel55-cHSF1, Adel55-HSP70, Adel55-HSP90 or Adel55-HSF1i. The treated tumor focus was surgically removed and tumor growth of the tumor focus on the opposite flank was assessed. In Figure 4, we show tumor growth curve of B16 (Figure 4A) or CT26 (Figure 4B) tumors over time, and the picture of the opposite flank of different group of mice bearing B16 or CT26 tumors at day 30 (Figure 4C, one mouse was chosen randomly from each group). It shows that Adel55-cHSF1 treatment of a single focus of multifocal tumors dramatically decreased tumor growth velocity of the secondary focus, and the growth is much slower than Adel55-HSP70 and Adel55-HSP90 treated group. However, PBS, Adel55 and Adel55-HSF1i treatment did not show such effect. It indicates that cHSF1 is a superior gene for neoadjuvant immunotherapy of various tumors.

## Discussion

Neoadjuvant immunotherapy is very important to prevent tumor relapse when the primary treatment method is surgical resection. Heat shock proteins are molecular chaperones previously identified to be the component of high molecular weight tumor cell lysate that conferred tumor immunity with tumor cells [23]. HSPs have the capability of binding antigenic peptides and presenting to antigen presenting cells to result in anti-tumor cellular immune responses [16]. Additionally, HSPs have been shown to activate antigen presenting cells to augment generation of immune responses [24]. Tumors engineered to overexpress HSPs have altered tumorigenic potential compared to wild type tumors [25]. In animal models, purified HSP-antigen complexes for vaccination have resulted in sustained anti-tumor immune responses against autologous tumor cells [26]. However, the immunity generated by the heat shock proteins, such as HSP70 or HSP90 was limited and only fifty percent of the mice treated could resist the secondary tumor challenge [27]. Consistent with these studies, our data also showed only 50–65% animals in the Adel55-HSP70 and Adel55-HSP90 treated group showed tumor resistance. To find a better gene and delivery method for enhanced neoadjuvant immunotherapy, we employed the oncolytic adenovirus vector to deliver constitutively active HSF1 gene. Our previous study showed cHSF1 delivery could eradicate tumor growth by increasing the oncolytic effect and replication of oncolytic adenovirus Adel55. We reasoned that the replication property of Adel55-cHSF1 could lead to higher gene expression than regular adenoviral vector and cHSF1 itself could lead to various heat shock protein gene expression, both of which are ideal for better cancer immunotherapy.

In our study, we used B16 and CT26 murine cancer cell line which are poorly immunogenic and tumor cell vaccination is incapable of generating anti-tumor immunity. We found that direct intra-tumor Adel55-cHSF1 injection prior to surgical resection had the capability of generating subsequent tumor resistance in 95% mice, which is much more advantageous than Adel55-HSP70 or Adel55-HSP90 injection. Cellular immune responses phenotypes were CD8+ T-cell mediated, which are consistent with reports in the literature in the murine tumor models. Interestingly, CTL reactivity was only detected in those mice treated with Adel55-cHSF1. No measurable CTL activity could be detected in PBS, Adel55 or Adel55-HSF1i treated groups. Adel55-cHSF1 was also effective in altering growth of distant tumor. In a model system with multiple tumor foci, animals with surgical resection of a single tumor focus injected with Adel55-cHSF1 continued to have immune responses and prevent the secondary tumor growth, and the velocity of tumor growth is much faster in Adel55-HSP70 and Adel55-HSP90 treated groups. It suggests Adel55-cHSF1 may be an effective strategy for the treatment of tumors where there is a concern for relapse due to micrometastatic disease.

In our system, Adel55-cHSF1 was both oncolytic and superior in generation of anti-tumor immune responses. This virus has the capability of replicating in tumor cells, resulting in tumor cell death, in addition to cHSF1 expression allowing for various HSP overexpression and liberation of HSP-antigen complexes for immune system recognition. cHSF1 gene transfer is not dependent on knowledge of the tumor-specific antigen and should be capable of generating tumor-specific immune responses against most immunogenic solid tumors.

## Conclusions

Our results suggest that the advantage of oncolytic adenoviral vector, Adel55-cHSF1 for tumor gene therapy includes the enhanced oncolytic adenovirus replication and induction of tumor-specific immune response. It has the potent for the combined application of heat shock pathway and oncolytic adenovirus for tumor treatment in the future.

## Abbreviations

HSF1, Heat shock transcription factor 1; cHSF1, Constitutively active heat shock transcription factor 1; HSF1i, HSF1 miRNA inhibitor; HSP, Heat shock protein; pfu, Plaque formation unit; APC, Antigen-presenting cell.

## Competing interests

The authors declare that they have no competing interests.

## Authors’ contributions

RF carried out all the animal experiments and CTL assay. CW made all the plasmids and viruses. YW maintained all the cell lines. PR, HJ, and WH collaborated in CTL assay. PG, ZX, SH and QZ collaborated in animal experiments. YJ participated in the design of the study and collaborated in the drafting the manuscript. XH was primarily responsible for designing of the study and drafting the manuscript. All authors have read and approved the final manuscript.
